# Cumulative effective dose from recurrent CT examinations in Europe: proposal for clinical guidance based on an ESR EuroSafe Imaging survey

**DOI:** 10.1007/s00330-021-07696-1

**Published:** 2021-03-12

**Authors:** Guy Frija, John Damilakis, Graciano Paulo, Reinhard Loose, Eliseo Vano, European Society of Radiology (ESR)

**Affiliations:** 1 Université de Paris, 12 Rue de l’École de Médecine, 75006 Paris, France; 2 School of Medicine, University of Crete, 71003 Iraklion, Greece; 3 ESTESC-Coimbra Health School, Medical Imaging and Radiotherapy Department, Instituto Politécnico de Coimbra, Rua 5 de Outubro, S. Martinho do Bispo, 3046-854 Coimbra, Portugal; 4 Institute of Medical Physics, Hospital Nuremberg, Prof.-Ernst-Nathan-Str. 1, 90419 Nuremberg, Germany; 5 Radiology Department, Complutense University, 28040 Madrid, Spain; 6 Am Gestade 1, Vienna, Austria

**Keywords:** X-ray computed tomography, Radiation exposure, Surveys and questionnaires, Clinical audit, Clinical decision support systems

## Abstract

**Abstract:**

In recent years, the issue of cumulative effective dose received from recurrent computed tomography examinations has become a subject of increasing concern internationally. Evidence, predominantly from the USA, has shown that a significant number of patients receive a cumulative effective dose of 100 mSv or greater. To obtain a European perspective, EuroSafe Imaging carried out a survey to collect European data on cumulative radiation exposure of patients from recurrent computed tomography examinations. The survey found that a relatively low percentage of patients (0.5%) received a cumulative effective dose equal to or higher than 100 mSv from computed tomography, most of them having an oncological disease. However, there is considerable variation between institutions as these values ranged from 0 to 2.72%, highlighting that local practice or, depending on the institution and its medical focus, local patient conditions are likely to be a significant factor in the levels of cumulative effective dose received, rather than this simply being a global phenomenon. This paper also provides some practical actions to support the management of cumulative effective dose and to refine or improve practice where recurrent examinations are required. These actions are focused around increasing awareness of referring physicians through encouraging local dialogue, actions focused on optimisation where a team approach is critical, better use of modern equipment and the use of Dose Management and Clinical Decision Support Systems together with focused clinical audits. The proper use of cumulative effective dose should be part of training programmes for referrers and practitioners, including what information to give to patients.

**Patient summary:**

Radiation is used to the benefit of patients in diagnostic procedures such as CT examinations, and in therapeutic procedures like the external radiation treatment for cancer. However, radiation is also known to increase the risk of cancer. To oversee this risk, the cumulative effective dose (CED) received by a patient from imaging procedures over his or her life is important. In this paper, the authors, on behalf of EuroSafe Imaging, report on a survey carried out in Europe that aims to estimate the proportion of patients that undergo CT examinations and are exposed to a CED of more than 100 mSv. At the same time, the survey enquires about and underlines radiologists’ measures and radiology departments’ strategies to limit such exposure. Over the period of 2015–2018, respondents reported that 0.5% (0–2.72%) of patients were exposed to a CED of ≥ 100 mSv from imaging procedures. The background radiation dose in Europe depends on the location, but it is around 2.5 mSv per year. It is obvious that patients with cancer, chronic diseases and trauma run the highest risk of having a high CED. However, even if the number of patients exposed to ≥ 100 mSv is relatively low, it is important to lower this number even further. Measures could consist in using procedures that do not necessitate radiation, using very low dose procedures, being very critical in requiring imaging procedures and increasing awareness about the issue.

**Key Points:**

*• A relatively low percentage of patients (0.5%) received a cumulative effective dose from CT computed tomography equal to or greater than 100 mSv, in Europe, most of them having an oncological disease.*

*• There is a wide range in the number of patients who receive cumulative effective dose equal to or greater than 100 mSv (0–2.72%) and optimisation should be improved.*

*• Increasing the awareness of referring physicians through encouraging local dialogue, concrete actions focused on optimisation and development of dose management systems is suggested.*

## Introduction

The International Atomic Energy Agency (IAEA) held a Technical Meeting between 4 and 6 March 2019 on recurrent examinations [1]. The purpose of this meeting was to discuss data showing that a significant number of patients who had recurrent computed tomography (CT) examinations received a cumulative effective dose (CED) of 100 mSv or higher. These patients often had a high number of examinations over a short period of time. The data discussed at this meeting was predominantly focused on the USA and included a study [2] that collected data covering 324 hospitals. This study looked at approximately 2.5 million patients and found that 1.33% received a cumulative effective dose from CT of ≥ 100 mSv. It is not clear if these results reflect the practice of the institutions and countries where the data was collected, or if it is a more general global phenomenon. This phenomenon, however, is not new as it was highlighted several years ago [3, 4], as well as more recently [5], although its magnitude has still not been established.

This paper will outline the results from a questionnaire, developed by EuroSafe Imaging, that aimed to collect European data on cumulative radiation exposure of patients from recurrent CT examinations. These results will then be used as a basis for any further recommendations and research.

## Methodology

Data was collected through a web-based questionnaire from 20 November 2019 until 22 January 2020. The questionnaire was sent to a total of 605 health professionals, consisting of all institutions belonging to the EuroSafe Imaging Stars network, to the heads of European academic centres and to Working Group members of EuroSafe Imaging. Invitations to complete the questionnaire were sent by email on 20 November 2019, with a subsequent reminder email sent on 4 December 2019. The survey closed on 22 January 2020.

The questionnaire consisted of 16 questions (see Table 1), of which 3 were mandatory. Questions covering the number of patients who received CT examinations, as well as the number and type of patients who received CED ≥ 100 mSv patients, collected data on patients who received examinations between 2015 and 2018. Following the conclusion of the survey, responses were analysed.

**Table 1:** List of survey questions

List of survey questions	
1. Personal information (Title, first name, email, hospital name, department name, city)	
2. In which country are you based?	
3. What was the total number of patients who received CT examinations in your hospital in the following years? (2018, 2017, 2016, 2015)	
4. Do you have a dose tracking/management system in your hospital?	
5. What is the total number of patients who have had a cumulative affective dose (CED) ≥ 100 mSv?	
6. Please provide the numbers of adult patients, from the following categories, who have had a cumulative effective dose CED ≥ 100 mSv (oncologic disease, chronic disease, trauma, transplant, other conditions)	
7. Please provide the numbers of children, from the following categories, who have had a cumulative effective dose CED ≥ 100 mSv (oncologic disease, chronic disease, trauma, transplant, other conditions)	
8. Which action(s) for improving the process to justify recurrent scans would be most effective in reducing the rate of repeat examinations? • Development and implementation of updated guidelines for cumulative doses • Implementation of alerts that take into account the number of previous examinations • Providing information about previous imaging exams to referrers and practitioners • Use of dose management systems • Othes (please specify)	
9. Which action(s), relating to optimisation of examinations, do you think would be most effective in reducing cumulative dose? • Low-dose and ultra-low dose exams, especially in repeat investigstions • Effective and transparent dose management • Introducing monitoring and recording tools to standardise the documentation of patient dose in electronic health records. • Developing non-ionising imaging investigations • Clinical audit tools • Other (please specify)	
10. Is dose management mandatory in your country?	
11. Do you think dose management should be mandatory in your country?	
12. Please select which of the following parameters are required for reporting CT examinations: CTDIvol, DLP, Effective dose, Other (please specify)	
13. Please select which of the following parameters are required for reporting interventional radiology procedures: Dose area product PKA, Entrance air kerma Ka, r, Effective dose, Other (please specify)	
14. Please select which of the following parameters are required for reporting diagnostic fluroscopy: Dose area product, PKA, Entrance air kerma Ka, r, Effective dose, Other (please specify)	
15. Please select which of the following parameters are required for reporting radiography: Dose area product PKA, Effective dose, Other (please specify)	
16. Please provide any comments you have about the survey.	

In question 2, respondents were asked in which country they are based. This question was answered by 80 respondents and skipped by 5. Of the 80 responses, 66 (82.5%) were from countries within the European Union and 14 (17.5%) from non-EU countries (see Table 2). Information to characterise the centres that responded was not recorded.

**Table 2:** Number of survey responses by country

Country	Inhabitants, in thousands, taken from UNs World Population Prospects [6]	Number of responses
Austria	9006	3
Belgium	11,590	4
Bulgaria	6948	2
Croatia	4105	3
Czech Republic	10,709	1
France	65,274	7
Germany	83,784	4
Greece	10,423	3
Hungary	9660	2
Ireland	4938	3
Israel	8656	1
Italy	60,462	14
Netherlands	17,135	1
Poland	37,847	1
Portugal	10,197	1
Romania	19,238	4
Serbia	8737	2
Slovenia	2079	2
Spain	46,755	9
Sweden	10,099	2
Switzerland	8655	5
Turkey	84,339	5
UK	67,886	1

In question 3, respondents were asked to provide the total number of patients who received CT examinations in their hospitals between 2015 and 2018. This question was answered by 60 respondents. Of these, 59 provided data for 2018, 57 for 2017, 54 for 2016 and 50 for 2015. The totals are outlined in Table 3.

**Table 3:** Total number of patients who received CT examinations by year, in the responsive institutions

Year	Total number of patients who received CT examinations
2015	1,387,867
2016	1,671,897
2017	1,796,195
2018	1,955,831
Total	6,811,790

## Results

The online questionnaire received a total of 85 responses, across 23 countries. The responses for question 1 are not included in these results as this question collected personal information about the respondents and their institution. The results for questions 13, 14 and 15 are not reported as they do not relate to CT examinations.

### Question 4—do you have a dose tracking/management system in your hospital?

**Fig. 1:**
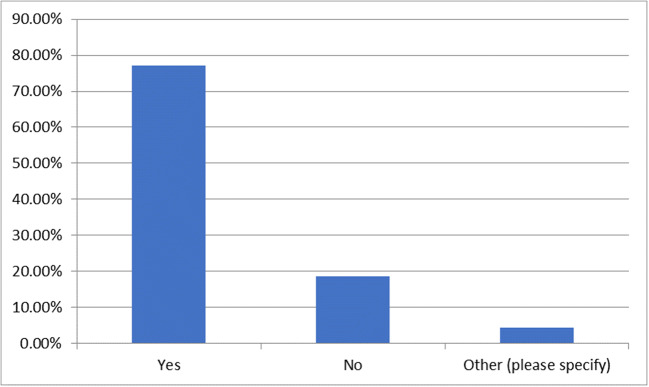
Percentage of respondents that have a dose tracking/management system in their hospital

This question received 70 responses and was skipped by 15. Of those who responded, 53 (75.7%) indicated that they have a dose tracking/management system in their hospital, 14 (20%) do not and 3 (4.3%) answered ‘other’ (Fig. 1). The 3 respondents that answered other all indicated that they had some form of dose tracking; one indicated that they had limited tracking capabilities, one had a newly installed dose management system (DMS) that is not yet fully utilised and another indicated that they have a self-developed dose management system.

### Question 5—what percentage of patients, who have had recurrent CT examinations, received a cumulative effective dose (CED) ≥ 100 mSv?

**Fig. 2:**
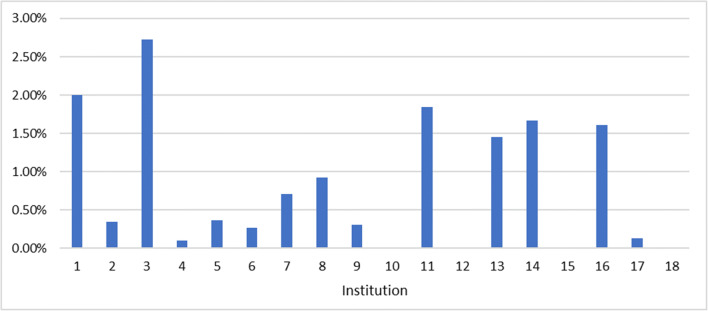
Percentage of patients, by institution, who have had recurrent CT examinations, who received a cumulative effective dose (CED) ≥ 100 mSv

This question received 18 responses, from individual institutions, covering a total of 1,218,429 patients, of which 6082 received a cumulative effective dose of (CED) ≥ 100 mSv. The percentage of patients who have received a cumulative effective dose (CED) ≥ 100 mSv, over the period of 2015–2018, ranges from 0 to 2.72%. The mean is 0.5% (Fig. 2).

Of those who responded to this question, 15 had a DMS, 2 indicated ‘other’ and 1 did not specify. For those that answered other, 1 indicated they had a self-developed DMS and the other that they had limited dose tracking. In terms of geographical distribution, the responses to this question came from the following countries: 2 from France, 1 from Germany, 5 from Italy, 3 from Spain, 1 from the UK, 3 from Turkey, 1 from Austria and 2 did not report their country. Additionally, of the 18 responses, 12 came from academic hospitals, 3 from general hospitals and 3 did not provide this data.

### Question 6—please provide the numbers of adult patients, from the following categories, who have had a cumulative effective dose (CED) ≥ 100 mSv

**Fig. 3:**
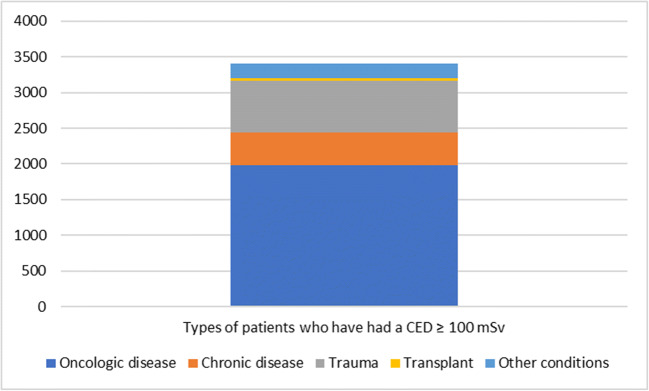
Categories of adult patients who have had a cumulative effective dose (CED) ≥ 100 mSv

This question was answered by 15 respondents, with the data covering a total of 903,336 patients, of which 3405 patients had a cumulative effective dose (CED) ≥ 100 mSv between 2015 and 2018 (Fig. 3). Of these patients, 1976 had an oncologic disease, 466 had a chronic disease, 718 were trauma patients, 43 had a transplant and 202 were indicated as ‘other conditions’ (which included 22 follow-up of post bariatric surgery, 16 cardiac complications and 135 post-operative complications; a further 29 were not specified).

### Question 7—please provide the numbers of children, from the following categories, who have had a cumulative effective dose (CED) ≥ 100 mSv

All 14 responses, corresponding to 903,365 patients who received CT scans, reported that no paediatric patients, for any of the categories, received CED ≥ 100 mSv. The available categories were oncologic disease, chronic disease, trauma patients, transplant and other.

### Question 8—which action(s) for improving the process to justify recurrent scans would be most effective in reducing the rate of repeat examinations?

**Fig. 4:**
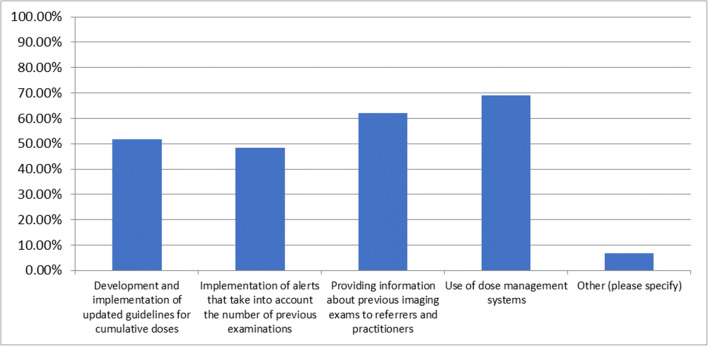
Which action(s) for improving the process to justify recurrent scans would be most effective in reducing the rate of repeat examinations?

This question was answered by 31 respondents and skipped by 54. Of the responses, 16 (51.6%) selected development and implementation of updated guidelines for cumulative doses, 16 (51.6%) selected implementation of alerts that take into account the number of previous examinations, 21 (67.7%) selected providing information about previous imaging exams to referrers and practitioners, 22 (71.0%) selected use of dose management systems and 3 (9.7%) selected other (Fig. 4).

The responses for other were that every CT scan needs to be justified regardless of prior exams and it would therefore be dangerous to withhold examinations because a patient had another CT before and 2 responses were that the routine use of clinical decision support tools would help.

### Question 9—which action(s), relating to the optimisation of examinations, do you think would be most effective in reducing cumulative dose?

**Fig. 5:**
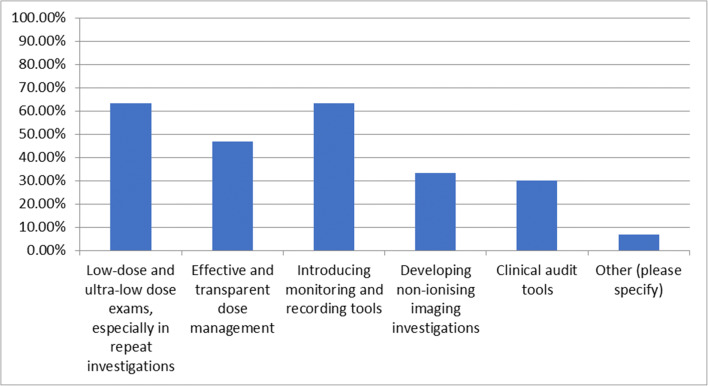
Which action(s), relating to the optimisation of examinations, would be most effective in reducing cumulative dose?

This question was answered by 32 respondents and skipped by 53. Of the responses, 21 (65.6%) selected low-dose and ultra-low dose exams especially in repeat investigations, 15 (46.9%) selected effective and transparent dose management, 20 (62.5%) selected introducing monitoring and recording tools to standardise the documentation of patient dose in electronic health records, 12 (37.5%) selected developing non-ionising imaging investigations, 10 (31.25%) selected clinical audit tools and 2 (6.25%) selected other. For those who answered other, one suggested that CT protocol management would be effective; the other commented that changing dose based on previous examinations per se is the wrong approach as if the dose can be lowered, it must be lowered, regardless of repeat examinations (Fig. 5).

### Question 10—is dose management mandatory in your country?

This question was answered by 35 respondents, with 18 (51%) answering that dose management is mandatory in their country and 17 (49%) answering that it is not mandatory.

### Question 11—do you think dose management should be mandatory in your country?

This question was answered by 33 and skipped by 52. All 33 (100%) respondents answered that they think dose management should be mandatory in their country (the responses came from the following countries: 2 from Austria, 1 from Belgium, 3 from Croatia, 1 from the Czech Republic, 3 from France, 3 from Germany, 1 from Hungary, 1 from Ireland, 5 from Italy, 1 from the Netherlands, 1 from Portugal, 4 from Spain, 1 from Switzerland, 2 from Turkey, 1 from the UK; a further 3 responses did not indicate their country).

### Question 12—please select which of the following parameters are required for reporting CT examinations

**Fig. 6:**
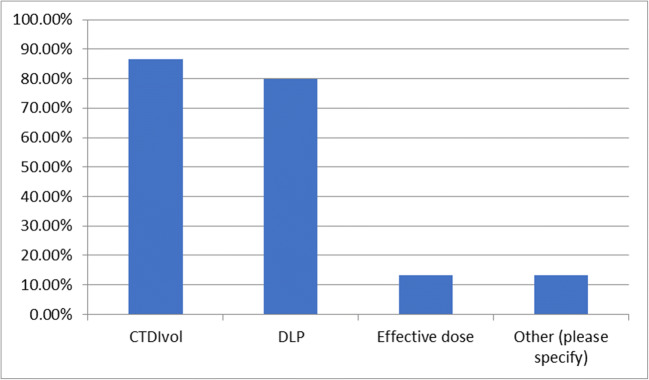
Parameters required for reporting CT examinations in responding institutions

Question 12 was answered by 17 respondents and skipped by 68. Of which, 14 (82.4%) selected CTDIvol, 14 (82.4%) selected DLP, 2 (11.8%) selected effective dose and 2 (11.8%) selected other (Fig. 6). The responses for those that answered other included the number of irradiation events.

### Question 16—please provide any comments you have about the survey

Three respondents commented that they were unable to provide any information on the number of patients who received CED ≥ 100 mSv, of which 2 indicated this was due to a lack of a dose management/tracking system to record this data.

## Discussion

The results of this study show that there was an increase in the number of patients who received CT examinations by about 41% between the years 2015–2018. This is partly due to the survey receiving an increasingly higher number of responses for more recent years. There has been a steady growth in the use of CT worldwide. A recent report [7] shows that in the USA, there was an estimated 25% increase in the number of CT scans performed in 2016 in comparison with those carried out in 2006 (about 67 million CT scans in 2006 compared to about 84 million in 2016). This increase may be attributed, at least partly, to improvements in CT technology and the widespread availability of CT systems. CT is a relatively high-dose modality with an estimated annual individual effective dose from CT at about 1.4 to 1.5 mSv [7]. For this reason, attention should be paid to the patients undergoing multiple CT exams resulting in cumulative effective dose of 100 mSv or more.

In this study, carried out amongst European facilities, we have shown that, between 2015 and 2018, a mean of 0.5% of patients received a CED ≥ 100 mSv, far less than that in a previous published study [2] (1.33%), which was mostly based on US American data. However, our study showed a wide range (from 0 to 2.72%) of CED ≥ 100 mSv, likely due to a large variation in practice across the centres (10 centres had less than 0.5% of patients who had recurrent CT examinations receiving CED ≥ 100 mSv and 8 higher than 0.5%). It is unclear whether this is related to the local protocols of these institutions or to differences in disease severity.

The comparison with American data is difficult because reported American DRLs [8] are higher than those established as part of the recent EU-funded EUCLID study for similar indications [9]. With the exception of stroke and appendicitis, the difference is between 30 and 500% more. This shows that the patient CT dose descriptors and estimated effective doses of recurrent examinations can be related to the protocols and not only to the number of examinations. It also remains unclear whether high CED was related to a high number of examinations or to a lower number with protocols using high exposure parameters.

This study provides insights on the diseases which are more commonly the subject of recurrent exposures. Oncology is the most predominant cause of CED from CT ≥ 100 mSv, followed by trauma and then chronic diseases.

The relevance of risks related to CED ≥ 100 mSv should be carefully considered, especially in the context of oncology, where the most important point from a patient perspective is the monitoring of the treatment efficacy, which is based today on recurrent CT examinations. In this context, an alarming warning should be avoided because RECIST biomarkers which are driving oncological treatments are determined with CT. In addition, almost every new drug requires a specific protocol and in this context the justification process application is rather limited. Ultra-low-dose protocols such as those for visualisation or attenuation correction in PET-CT should be handled with care in oncology and may become critical in case of RECIST follow-up [10]. The impact of high CED in this context would likely be low, as well the projected stochastic effects related to this.

Non-cancerous diseases require special attention [11–15] when imaging is recurrently used. In particular, where possible, the use of non-ionising radiation modalities should be explored and proposed to the physicians, for instance in chronic vascular disease [16]. More generally, the benefits and risks of recurrent examinations should be approached on a per-patient basis and we think decisions should be shared with the physicians in charge of the patients. This clearly goes beyond the development of any generic guidelines because local culture, practice and imaging access have to be considered, although some respondents suggested the development of specific guidelines in the context of recurrent examinations. More generally, we fear that 100 mSv could be seen as a constraint and therefore, a more practical approach based on the number of examinations would be preferable.

Implementation of the justification requirements of the European BSS [17] in daily practice is currently inconsistent across different countries and faces several challenges. It has been the European experience for many years that guidelines, even if available, have not been used. It is expected that point of care systems, like CDS (clinical decision support), would help to increase the use of guidelines and it might also be anticipated that use of CDS would ease the development of localised guidelines [18–20].

The survey showed the use of Dose Management Systems (DMS) should be very useful in the context of detecting patients that are the subject of recurrent examinations and may help with their management [21]. Alerts in the DMS may be used as an indication to review if the available referral criteria are being properly applied. Of the centres surveyed, DLP and CTDI are principally used and calculation of CED by DMS remains an issue as only some systems are capable of calculating this automatically. Manual tracking would be possible, in theory, but would be cumbersome and be a source of potential errors and therefore cannot be recommended.

In Europe, clinical audit is also considered to be an effective tool for reviewing the routine use of the justification process [17] and could be focused on the management of patients undergoing recurrent examinations.

Finally, recommending that imaging centres identify the patient categories with a greater likelihood of undergoing recurrent examinations is certainly an important step. This is because the optimisation of procedures remains, in this context, a major task, even if optimisation has to be achieved for every procedure. The awareness of physicians should be developed in parallel and agreed examination planning should be sought in a multidisciplinary setting, not forgetting the use of non-ionising radiation modalities. Shared optimisation strategies for patients with CED ≥ 100 mSv should be sought with the physicians in charge of the patients. This study supports the idea that recurrent exposures should be included in the scope of further clinical audit and potentially of regular inspections, at least in Europe [17]. The ESR’s Clinical Audit tool, Esperanto [22], includes relevant audit templates. In particular, template 15 covers mechanisms used to evaluate patient dose in high-dose procedures.

Even if it is expected from industry to provide sub-millisievert CT examinations, it is a matter of fact that a good use of technology and appropriate guidance of serial examinations will remain the key for controlling the recurrent examination burden.

The magnitude of patients receiving CED ≥ 100 mSv still needs to be better determined as it appears that the figures are dependent on the setting and available technology. This means that the magnitude is most likely related to local practice. This survey did not collect relevant information on centres’ patient recruitment or information on available technology, which could explain differences between centres having either a low or a high number of patients with CED ≥ 100 mSv. Existing European regulation addresses the issue of justification and optimisation and we are convinced that localised guidelines should be developed instead of generic ones, in addition to the use of optimised protocols, the use of DMS and the use of clinical audit.

This study is based on a large number of responses but it should be noted that only a limited number of responses (18 from 85) gave data on the percentage of patients with cumulative effective doses and therefore has to be complemented by a wider study in order to better assess the European magnitude. However, our study shows that the use of DMS would be critical for successfully carrying out this wider study. Paediatric patients who could be subject to recurrent examinations [23] were not reported in the study; dose management systems were not used by all centres that responded, patient age data was not required in the survey and the method of calculation of effective dose was not assessed.

Nevertheless, even if the number of respondent centres is low, the number of examinations considered is rather high (1,218,429 patients): thus, this study provides relevant qualitative information with enough material for proposing some practical actions, as stated in Table 4, regardless of the magnitude of the CED phenomenon. Our understanding of the magnitude of CED relies for the moment on one large study performed in the USA, which we do not think could be simply extrapolated to Europe but we think it contributes to the justification of the proposed actions.

**Table 4:** How to reduce the number of recurrent examinations?

Having discussions with the physician requester	
✓ Highlighting the potential risk/benefit of recurrent examinations	
✓ Discussing whether decreasing the number of exams and/or replacing CT with MRI or Ultrasound would be possible	
✓ Informing them that they would be provided with the cumulative dose for each patient having recurrent CT examinations	
Developing actions	
✓ Establish the list of clinical situations where recurrent CT examinations are undertaken in the institution	
✓ CT Protocol optimisation to perform the procedure at the lowest dose for the clinical indication	
✓ Involve the radiographers and the medical physicists of the department and increase awareness	
✓ Set a dose tracking system for these patients, ideally integrated into the electronic health record	
✓ Provide the physician requester with feedback on the cumulative dose reached for each concerned patient in order to constantly update the benefit–risk estimation	
✓ Set-up an internal audit focused specifically on patients undergoing recurrent examinations	
✓ Develop localised guidelines on patient follow-up when the CED ≥ 100 mSv	

## Conclusion

Recurrent CT examinations are a common feature in some specific clinical situations, which require specific clinical solutions.

Oncology follow-up, chronic diseases, transplants, trauma, and cardiovascular diseases are the most common situations for recurrent examinations in adults.

The cumulative dose in these clinical situations could reach levels that according to ICRP [24] increase the risk of complications related to high dosage. Although this occurs in a very low percent of patients, specific attention should be given in order to minimise any negative effects, particularly in optimising the good use of imaging modalities.

The use of DMS is considered one critical tool but increasing the awareness of physicians also looks to be critical to improve management of recurrent examinations safety. The proper use of CED should be included in training programmes for referrers and practitioners (including information for patients).

## Abbreviations

CED: Cumulative effective dose
CT: Computed tomography
DLP: Dose length product
DMS: Dose management systems

## References

[1] Brambilla M, Vassileva J, Kuchcinska A, Rehani M (2020). Multinational data on cumulative radiation exposure of patients from recurrent radiological procedures: call for action. Eur Radiol.

[2] Rehani MM, Yang K, Melick ER (2019). Patients undergoing recurrent CT scans: assessing the magnitude. Eur Radiol.

[3] Durand DJ, Dixon RL, Morin RL (2012). Utilization strategies for cumulative dose estimates: a review and rational assessment. J Am Coll Radiol.

[4] Walsh L, Shore R, Auvinen A, Jung T, Wakeford R (2014). Risks from CT scans–what do recent studies tell us?. J Radiol Prot.

[5] Vano E (2020). Recurrent imaging procedures with ionising radiation on the same patient. Should we pay more attention?. J Radiol Prot.

[6] United Nations World Population Prospects (2019) Available via https://population.un.org/wpp/Download/Standard/Population/. Accessed 23 Sep 2020

[7] National Council on Radiation Protection and Measurements (2019). Medical radiation exposure of patients in the United States, NCRP report 184.

[8] Smith-Bindman R, Wang Y, Chu P (2019). International variation in radiation dose for computed tomography examinations: prospective cohort study. BMJ.

[9] European Commission Tender Project EUCLID – European Study on Clinical Diagnostic Reference Levels for X-ray Medical Imaging (2017). Available via http://www.eurosafeimaging.org/euclid. Accessed 23 Sep 2020

[10] Eisenhauer EA, Therasse P, Bogaerts J (2009). New response evaluation criteria in solid tumours: revised RECIST guideline (version 1.1). Eur J Cancer.

[11] Rehani MM, Melick ER, Alvi RM (2020). Patients undergoing recurrent CT exams: assessment of patients with non-malignant diseases, reasons for imaging and imaging appropriateness. Eur Radiol.

[12] Brambilla M, De Mauri A, Leva L, Carriero A, Picano E (2013). Cumulative radiation dose from medical imaging in chronic adult patients. Am J Med.

[13] De Mauri A, Brambilla M, Izzo C (2012). Cumulative radiation dose from medical imaging in kidney transplant patients. Nephrol Dial Transplant.

[14] Desmond AN, McWilliams S, Maher MM, Shanahan F, Quigley EM (2012). Radiation exposure from diagnostic imaging among patients with gastrointestinal disorders. Clin Gastroenterol Hepatol.

[15] Brambilla M, Cerini P, Lizio D, Vigna L, Carriero A, Fossaceca R (2015). Cumulative radiation dose and radiation risk from medical imaging in patients subjected to endovascular aortic aneurysm repair. Radiol Med.

[16] Manning BJ, O’Neill SM, Haider SN, Colgan MP, Madhavan P, Moore DJ (2009). Duplex ultrasound in aneurysm surveillance following endovascular aneurysm repair: a comparison with computed tomography aortography. J Vasc Surg.

[17] European Council Directive 2013/59/Euratom on basic safety standards for protection against the dangers arising from exposure to ionising radiation (2014). Official Journal of the European Union L13;57:1–73 Available at https://eur-lex.europa.eu/LexUriServ/LexUriServ.do?uri=OJ:L:2014:013:0001:0073:EN:PDF

[18] Centers for Medicare & Medicaid Services (CMS), Department of Health and Human Services (HHS) (2016) Appropriate Use Criteria Program.https://www.cms.gov/Medicare/Quality-InitiativesPatient-Assessment-Instruments/Appropriate-Use-CriteriaProgram/index.html. Accessed 7 Nov 2019

[19] Moriarity AK, Klochko C, Obrien M, Halabi S (2015). The effect of clinical decision support for advanced inpatient imaging. J Am Coll Radiol.

[20] Palen TE, Sharpe RE, Shetterly SM, Steiner JF (2019). Randomized clinical trial of a clinical decision support tool for improving the appropriateness scores for ordering imaging studies in primary and specialty care ambulatory clinics. AJR Am J Roentgenol.

[21] Loose RW, Vano E, Mildenberger P et al. Dose management – requirements and recommendations for users. EuroSafe Imaging 2020 / ESI-11078. 10.26044/esi2020/ESI-11078 Available via https://epos.myesr.org/poster/esr/eurosafeimaging2020/ESI-11078 Accessed 27 Jun 2020

[22] The ESR Clinical Audit booklet Esperanto (2019) Available via https://www.myesr.org/media/2835. Accessed 13 Oct 2020

[23] Brambilla M, De Mauri A, Lizio D (2014). Cumulative radiation dose estimates from medical imaging in paediatric patients with non-oncologic chronic illnesses. A systematic review. Phys Med.

[24] The 2007 Recommendations of the International Commission on Radiological Protection. ICRP Publication 103. Ann ICRP 37(2–4):1–332 10.1016/j.icrp.2007.10.003 18082557

